# Supplementary material for: Resolving the full spectrum of human genome variation using Linked-Reads
Source: Genome Res. 2019 Apr;29(4):635–45. doi: 10.1101/gr.234443.118 (PMC6442396; doi:10.1101/gr.234443.118)
Supplement: Supplemental Material [file supp_gr.234443.118_Supplemental_longranger-2.2.zip › longranger-2.2.tar.bz2!/lr2.2-public/lib/stan/cmdstan/stan/doc/api/html/check__positive__ordered_8hpp_source.html]

Stan: src/stan/math/error\_handling/matrix/check\_positive\_ordered.hpp Source File


|  |  |
| --- | --- |
|  | Stan  2.5.0  probability, sampling & optimization |


- Main Page
- Related Pages
- Namespaces
- Classes
- Files

- File List
- File Members

All Classes Namespaces Files Functions Variables Typedefs Enumerations Enumerator Friends Macros Pages

- src
- stan
- math
- error\_handling
- matrix

check\_positive\_ordered.hpp

Go to the documentation of this file.

1 #ifndef STAN\_\_MATH\_\_ERROR\_HANDLING\_\_MATRIX\_\_CHECK\_POSITIVE\_ORDERED\_HPP

2 #define STAN\_\_MATH\_\_ERROR\_HANDLING\_\_MATRIX\_\_CHECK\_POSITIVE\_ORDERED\_HPP

3

4 #include <sstream>

5 #include <stan/math/error\_handling/dom\_err.hpp>

6 #include <stan/math/matrix/Eigen.hpp>

7 #include <stan/math/matrix/meta/index\_type.hpp>

8 #include <stan/meta/traits.hpp>

9

10 namespace stan {

11

12  namespace math {

13

28  template <typename T\_y, typename T\_result>

29  bool check\_positive\_ordered(const char\* function,

30  const Eigen::Matrix<T\_y,Eigen::Dynamic,1>& y,

31  const char\* name,

32  T\_result\* result) {

33  using Eigen::Dynamic;

34  using Eigen::Matrix;

35  typedef typename index\_type<Matrix<T\_y,Dynamic,1> >::type size\_type;

36  if (y.size() == 0) {

37  return true;

38  }

39  if (y[0] < 0) {

40  std::ostringstream stream;

41  stream << " is not a valid positive\_ordered vector."

42  << " The element at " << stan::error\_index::value

43  << " is %1%, but should be postive.";

44  std::string msg(stream.str());

45  return dom\_err(function,y[0],name,

46  msg.c\_str(),"",

47  result);

48  }

49  for (size\_type n = 1; n < y.size(); n++) {

50  if (!(y[n] > y[n-1])) {

51  std::ostringstream stream;

52  stream << " is not a valid ordered vector."

53  << " The element at " << stan::error\_index::value + n

54  << " is %1%, but should be greater than the previous element, "

55  << y[n-1];

56  std::string msg(stream.str());

57  return dom\_err(function,y[n],name,

58  msg.c\_str(),"",

59  result);

60  }

61  }

62  return true;

63  }

64

65  }

66 }

67 #endif

dom\_err.hpp

stan::math::dom\_err

bool dom\_err(const char \*function, const T &y, const char \*name, const char \*error\_msg, const T\_msg error\_msg2, T\_result \*result)

**Definition:** dom\_err.hpp:36

traits.hpp

stan::math::index\_type

Primary template class for the metaprogram to compute the index type of a container.

**Definition:** index\_type.hpp:21

stan::error\_index::value

**Definition:** traits.hpp:20

index\_type.hpp

Eigen.hpp

stan::math::size\_type

index\_type< Eigen::Matrix< double, Eigen::Dynamic, Eigen::Dynamic > >::type size\_type

Type for sizes and indexes in an Eigen matrix with double e.

**Definition:** typedefs.hpp:16

stan::math::check\_positive\_ordered

bool check\_positive\_ordered(const char \*function, const Eigen::Matrix< T\_y, Eigen::Dynamic, 1 > &y, const char \*name, T\_result \*result)

Return true if the specified vector contains only non-negative values and is sorted into increasing o...

**Definition:** check\_positive\_ordered.hpp:29


---

[ Stan Home Page ]

*© 2011–2014,
Stan Development Team.*
